# Interspecies Correlations between Human and Mouse *NR2E3*-Associated Recessive Disease

**DOI:** 10.3390/jcm10030475

**Published:** 2021-01-27

**Authors:** Alessandro Iannaccone, Emily Brabbit, Christiaan Lopez-Miro, Zoe Love, Victoria Griffiths, Marina Kedrov, Neena B. Haider

**Affiliations:** 1 Center for Retinal Degenerations and Ophthalmic Genetic Diseases, Department of Ophthalmology, Duke Eye Center, Duke University School of Medicine, Durham, NC 27710, USA; christiaan.lopezmiro@duke.edu (C.L.-M.); victoria.griffiths@duke.edu (V.G.); marina.kedrov@duke.edu (M.K.); 2 Schepens Eye Research Institute, Massachusetts Eye and Ear, Department of Ophthalmology, Harvard Medical School, Boston, MA 02114, USA; eabrabbit@gmail.com (E.B.); lovzoe@gmail.com (Z.L.)

**Keywords:** *NR2E3*, *rd7*, Enhanced S-Cone Syndrome, retinal degeneration, optical coherence tomography, autofluorescence, imaging, photoreceptor degeneration

## Abstract

*NR2E3*-associated recessive disease in humans is historically defined by congenital night blinding retinopathy, characterized by an initial increase in short-wavelength (S)-cone sensitivity and progressive loss of rod and cone function. The retinal degeneration 7 (*rd7*) murine model, harboring a recessive mutation in the mouse ortholog of *NR2E3*, has been a well-studied disease model and recently evaluated as a therapeutic model for *NR2E3*-associated retinal degenerations. This study aims to draw parallels between human and mouse *NR2E3*-related disease through examination of spectral domain optical coherence tomography (SD-OCT) imaging between different stage of human disease and its murine counterpart. We propose that SD-OCT is a useful non-invasive diagnostic tool to compare human clinical dystrophy presentation with that of the *rd7* mouse and make inference that may be of therapeutically relevance. Additionally, a longitudinal assessment of *rd7* disease progression, utilizing available clinical data from our patients as well as extensive retrospective analysis of visual acuity data from published cases of human *NR2E3*-related disease, was curated to identify further valuable correlates between human and mouse *Nr2e3* disease. Results of this study validate the slow progression of *NR2E3*-associated disease in humans and the *rd7* mice and identify SD-OCT characteristics in patients at or near the vascular arcades that correlate well with the whorls and rosettes that are seen also in the *rd7* mouse and point to imaging features that appear to be associated with better preserved S-cone mediated retinal function. The correlation of histological findings between *rd7* mice and human imaging provides a solid foundation for diagnostic use of pathophysiological and prognostic information to further define characteristics and a relevant timeline for therapeutic intervention in the field of *NR2E3*-associated retinopathies.

## 1. Introduction

Development of the neural retina is characterized by the mitotic division and terminal differentiation of pluripotent progenitor cells to produce several distinct classes of cell types in the retina (ganglion, amacrine, cone photoreceptors, bipolar, horizontal, rod photoreceptors, and Müller glia) [1,2,3,4,5,6,7,8,9]. Normal retinogenesis for the various cell types is maintained by both intrinsic transcriptional machinery and extrinsic mitogenic factors to ensure correct cell-fate specification [10,11,12,13,14,15]. Previous studies have shown that rod and cone photoreceptor differentiation is heavily regulated by the nuclear hormone receptor (NHR) superfamily, a group of transcription factors that aid in maintaining the homeostatic state of retinal cell processes [16,17,18]. The nuclear hormone receptor 2, family E, member 3, (*NR2E3),* functions with cofactors to promote rod cell differentiation and suppress the formation of cones [19,20,21,22,23,24,25,26]. Inherited mutations in *NR2E3* lead to a dramatic decrease in rod function and reduced long- to middle-wavelength sensitive (L/M) cone function, paired with marked increase in the normally least populous and less robust short-wavelength sensitive (S) cone function [22,27,28,29]. This dysregulation of rod development and consequential alteration of post-mitotic photoreceptor precursor cell fate gives rise to a variety of inherited retinal degenerative diseases that have been termed Goldmann-Favre Vitreoretinal Dystrophy (GFVRD) [30,31], enhanced S-cone syndrome (ESCS) [31,32,33,34], and clumped pigmentary retinal degeneration (CPRD) [35]. These allelic autosomal recessive conditions share the clinical characteristic of deep nummular changes at the arcades [36,37] and the functional characteristic of absolute or relative enhancement of S-cone mediated retina function [27,28,29,31,32,33,34,38,39,40,41].

The *retinal degeneration 7 (rd7)* murine model, *Nr2e3^rd7/rd7^* (*rd7*), lacks a functional *Nr2e3* gene and is a model for all *Nr2e3* associated retinal degenerations [24,42]. More recently, the *rd7* model has also been used to evaluate targeted gene therapy strategies due to its novel similarity in clinical phenotype to human patients with *NR2E3*-associated retinal degenerations [43]. *NR2E3* disease in the *rd7* mouse is characterized by two distinct phenomena: a developmental defect in photoreceptor development characterized by an increase in cone photoreceptor cells, and slow, progressive photoreceptor degeneration [24,44]. Over-proliferation of cone photoreceptor cells results in disruption of retinal topography. Interestingly, while retinal topography is disrupted, the distribution of rods and cones across the retina remains relatively normal. The *rd7* mouse exhibits whorls and rosettes in the outer nuclear layer (ONL) of the retina, which are thought to correlate to their clinical phenotype of pan-retinal spotting shown through fundus imaging [24]. As *Nr2e3* disease advances, a progressive degeneration of rods and cones results in the resolution of the whorls and rosettes and concomitant fading of retinal spots in the *rd7* mouse fundus fade with disease progression.

Previous studies have outlined the following aging alignment of control C57Bl6/J (B6) mice to that of a human individual: young (1–3 months), mature (3–6 months), middle-aged (10–14 months), and aged (18–24 months) mice and the human life phase equivalents (20–30 years, 38–47 years, and 56–69 years, respectively) [45]. The current clinical approach lacks an ample standard diagnostics protocol for disease characterization and rate of progression in the human population. This study presents a maturational rate comparison between human and mouse *NR2E3*-associated disease in order to not only better understand the meaning of certain unique characteristics of human imaging findings, but to also evaluate and compare stages of *NR2E3*-associated disease characteristics between species to consequentially provide insight into an ideal time for therapeutic intervention. Herein, we illustrate a number of fundamental phenotypic analogies between the *rd7* murine phenotype and that observed in human patients expressing the ESCS phenotype as seen through ophthalmoscopy and spectral domain optical coherence tomography (SD-OCT). Furthermore, we present functional correlates to certain SD-OCT characteristics and findings that suggest a likely predictive correlation with better preserved S-cone mediated retinal function. By virtue of these function-structure correlations, we propose that SD-OCT can allow for an informative, noninvasive assessment of *NR2E3*-associated retinal disease in live human patients.

## 2. Materials and Methods

### 2.1. Ethics Statement

All clinical examinations of affected patients were conducted in full compliance with the Declaration of Helsinki in a tertiary patient care setting (Duke Center for Retinal Degenerations and Ophthalmic Genetic Diseases) according to standard diagnostic procedures. No human test or examination was conducted solely for the purpose of this retrospective research analysis. Genotyping was performed on all patients via Clinical Laboratory Improvement Amendments (CLIA)-certified molecular genetic diagnostic testing in a standard clinical setting with written consent from all patients, including parental consent for the minors, as well as on all parents (when available) to confirm the phase of the detected mutations. This retrospective analysis was conducted with the approval of the Duke Institutional Review Board under Protocol 00092003.

### 2.2. Clinical Studies

Five patients (PT) (2 males, 3 females; age range: 9–69 years old (yo), mean ± 1SD: 34 ± 29 yo; 3 Caucasian subjects, one of Italian and two of mixed Northern European descent, and 2 African siblings, both of Ethiopian descent) harboring homozygous or compound heterozygous *NR2E3* mutations resulting in an ESCS phenotype have been retrospectively evaluated. The salient patient characteristics, including their genotypes, are summarized in Table 1.

Three of the patients were in the pediatric age range (9–16 yo) and the other two were adults (61 and 69 yo, respectively). For the purpose of comparison between the human and the *rd7* murine phenotype, these age ranges corresponded to mouse age ranges between 1 and 3 months and 18–24 months, respectively. The indicated ages in Table 1 refer to the most recent (latest) examinations. For the first of the two adult patients, we also had access to retrospective longitudinal clinical and perimetric data available spanning several decades.

Functional examinations were obtained according to previously published procedures and methods [37,46,47] and included best-corrected visual acuity (BCVA) to Early Treatment of Diabetic Retinopathy Study (ETDRS) charts, kinetic perimetry (performed either with a manual Goldmann perimeter or with an Octopus 900 Pro semi-automated kinetic perimeter, Haag-Streit Diagnostics USA, Mason, OH), automated static chromatic perimetry [performed with size-V stimuli to dark- (DA) and light-adapted (LA) stimuli to separate the contributions of rods (DA 500 nanometer (nm)), L/M-cones (LA 600 nm), mainly L-cones (LA 650 nm), and S-cones (LA 440 nm on a bright yellow background) using either a custom-modified Humphrey Field Analyzer (HFA) as previously reported [37,47] or a standard HFA offering the short wavelength automated perimetry (SWAP) option], full-field flash electroretinograms (ffERGs) recorded with an Espion3 unit (Diagnosys LLC, Lowell, MA, USA) in response to DA and LA stimuli in line with the ISCEV standards [48].

Information on the spontaneous fluorescence characteristics originating from the retinal pigment epithelium (RPE) and the clinical appearance of the patient fundus were obtained via wide-field fundus autofluorescence (WF-FAF) and color fundus photography, respectively (all with wide field imaging (Optos, Marlborough, MA, USA), except for PT1 and PT4 for whom a Zeiss color fundus camera was used instead (Carl Zeiss Meditec, Inc., Dublin, CA, USA). The characteristics of retinal microanatomy in the pericentral areas were evaluated by in vivo imaging via standard spectral domain optical coherence tomography scans (SD-OCT, Heidelberg Engineering USA, Franklin, MA, USA) performed at custom locations, at or near the vascular arcades. In PT5, we were also able to obtain wide-field (WF) SD-OCT scans with the same instrument at her last exam. The human SD-OCT retinal scans were compared to corresponding in vivo OCTs findings of *rd7* mice. Examples of kinetic perimetry, fundus appearance, FAF, and OCT scans in a normal subject are provided in Figure S1.

### 2.3. Animal In Vivo Studies

Animal studies were carried out in strict accordance with the recommendations in the Guide for the Care and Use of Laboratory Animals of the National Institute of Health. Animal use and procedures were approved by the Schepens Eye Research Institute Animal Care and Use Committee (Protocol Numbers: S-477-0320, S-485-0620 and S-556-0323) in compliance with the Animal Welfare Act Regulations. All efforts were made to minimize animal suffering.

Animals used in this study were housed and bred in the vivarium at the Schepens Eye Research Institute under standard conditions. C57BL/6J (B6) (Jax stock # 000664) and *rd7* mice (Jax stock #004643) have been bred in our lab for the last two decades. Jax mice are bred with our strains every 1–2 years to ensure we do not have any long-term genetic isolation events that can create a subline. All studies were performed on mice at 1, 3, 6, and 12–18 months age matched B6 (control) or *rd7* mice. There are no observable gender differences due to *Nr2e3* mutations, thus both males and females were used equally. N denotes the number of biological variables in each study. 

Fundus and OCT were performed as previously described [43,49,50]. Mice were anesthetized using a mixture of Ketamine (100–200 miligram/kilogram body weight (mg/kg) given intraperitoneally (i.p.)) and Xylazine (20 mg/kg i.p.) using a 0.5′ 27-gauge needle for both indirect ophthalmoscopy and OCT. Both right and left eyes were dilated in a single topical dose using 1% tropicamide solution. Fundus images were taken via indirect ophthalmoscopy using a Micron III Retinal Imaging Camera and Stream Pix software (Phoenix Research Laboratories, Pleasanton, CA, USA). Following fundus imaging, OCT was performed using the SD-OCT scanner Imaging System (Envisu R2200, Bioptigen, Morrisville, NC, USA). Mice were restrained in a mounting tube and the fundus camera in the optical head of the apparatus and alignment was guided by monitoring and optimizing the real time OCT image of the retina. Four rotational cross section scans (dorsal-ventral and nasal-caudal) with 100 series/scan were taken near the central retina for each animal. Data were analyzed using the Bioptigen OCT software. N = 5/strain.

### 2.4. Animal Histology and Immunohistochemistry

Following euthanasia of animals, eyes were cauterized to mark dorsal orientation and enucleated. Tissue samples were collected and immediately immersed in freshly prepared 4% paraformaldehyde (PFA) in 1x phosphate buffered solution (PBS) or in 3:1 methanol/acetic acid overnight at 4 °C. Eyes were then paraffin embedded with dorsal/ventral orientation and 5 micrometer (µm) sections were collected over 100 µm of retinal depth and were then processed for hematoxylin/eosin (H/E) staining or immunohistochemistry as previously described [43,49,50,51]. For H/E staining, retina sections were deparaffinated in xylene and ethanol washed before being stained with hematoxylin and eosin Y. Slides were mounted with Permount mounting medium (Vector Laboratories). Sections were analyzed and imaged with the Leica DMI6000 microscope. Outer nuclear layer (ONL) cell counts were performed on serial sections of *rd7* and B6 control, counting a vertical line of nuclei within a typical whorl. Images were taken at 20× magnification and analyzed in a double-blinded manner over 100μm retinal area. Statistical significance of differential ONL cell count between B6 control and *rd7* was assessed using a *T*-test and *p* value of <0.05. Results are mean ± SEM (N  =  5).

Immunohistochemistry was performed using, at minimum, 100 µm of retina/sample. Sections for opsins labeling were blocked with 2% normal horse serum (#S-2000 VectorLabs, Burlingame, CA, USA) in PBS and incubated with the following cell type-specific primary antibodies at 1:200 dilution: rhodopsin (mouse monoclonal, Sigma Millipore MAB5316, Temecula, CA, USA); green/red opsin (rabbit polyclonal, Sigma Millipore AB5405, Billerica, MA, USA); or blue opsin (rabbit polyclonal, Sigma Millipore AB5407, Billerica, MA, USA). Sections for Iba1 labeling were blocked with 2% normal horse serum and 0.01% TritonX100 and incubated with the following cell type-specific primary antibody at 1:200 dilution: recombinant anti-Iba1 antibody (rabbit monoclonal, Abcam ab178847, Cambridge, UK). The following day, all sections were rinsed with PBS and incubated with the corresponding secondary antibody (1:400 Alexa fluor 488 goat antirabbit, Invitrogen A11008 for blue opsin, green/red opsin, and Iba1 staining; 1:400 Alexa fluor 488 goat anti-mouse, Invitrogen A1101 for rhodopsin staining, Invitrogen: Thermo Fisher Scientific, Waltham, MA, USA) and nuclei were labeled with 4,6-Diamidino-2-Phenylindole, Dihydrochloride (DAPI, 10236276001, Sigma Millipore, Milwaukee, WI, USA). Over 100 micrometer (μm) of sections/animal were visualized and representative images were captured using a Leica DMI6000 fluorescent microscope equipped with the appropriate bandpass filter for each fluorochrome. Iba1 cell counts were performed on serial sections of *rd7* and B6 control. Images were taken at 10× magnification and analyzed via ImageJ over 800 μm retinal area. Statistical significance of differential Iba1 cell count between inner and outer retinas of B6 control and *rd7* was assessed using a *T*-test and *p* value of <0.05. Results are mean ± SEM (N  =  5).

## 3. Results

### 3.1. Clinical and Imaging Findings in ESCS Patients

The basic demographic and genetic characteristics of our 5 patients are summarized in Table 1. All patients had night blindness noted since early childhood. Their age range varied widely and, as we will illustrate, is well representative of the various ESCS disease stages that have been recently proposed [39]. In each case, the typical ESCS functional phenotype was confirmed in full by ffERG criteria and/or by chromatic perimetry criteria. BCVA was variable, but in general, only mildly reduced (Mean ± standard deviation (SD): 0.67 ± 0.19; range: 0.40–1.00, i.e., between 20/50 and 20/20) and the worst case—PT1, the youngest—reflected a refractive amblyopic component. Except for this particular instance, all patients retained at least 20/30 in the better-seeing eye as late as age 69 yo.

**Figure 1:**
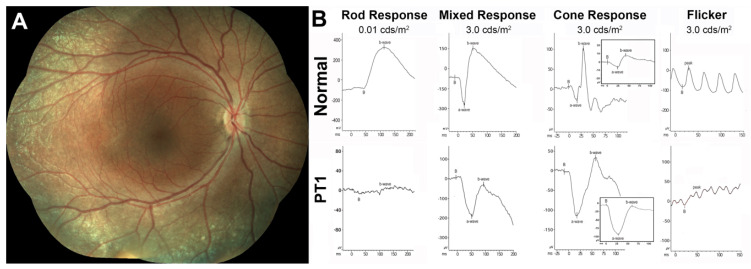
Clinical, functional, and imaging presentation in pediatric patient 1 (PT1). (**A**) Color fundus photography composite from the right eye of PT1; (**B**) Full-field flash ERG (ffERG) responses of PT1 compared to a normal example. S-cone selective ffERG responses are shown in the inset. Flash intensities for the ffERGs are shown in standard measuring units (candelas-second/square meter, cds/m^2^).

*Patient 1* (*PT1*). The clinical and functional phenotype exhibited by PT1, the youngest in our case series, is illustrated in Figure 1.

PT1 presented at age 9 yo only with some whitish fleck-like nummular deep deposits outside the arcades (Figure 1A). She was too young to undergo reliable perimetries, and no FAF images could be collected. At this stage of the disease, the mixed ffERG response exhibited the typical ESCS electronegativity with a normal a-wave and a markedly supernormal and electronegative cone transient response, driven mainly by S-cones (Figure 1B). This finding predicts, albeit by ffERG criteria and not by perimetric ones, that she likely met criteria for Stage 1 disease, characterized by supernormal S-cone function [39].

**Figure 2:**
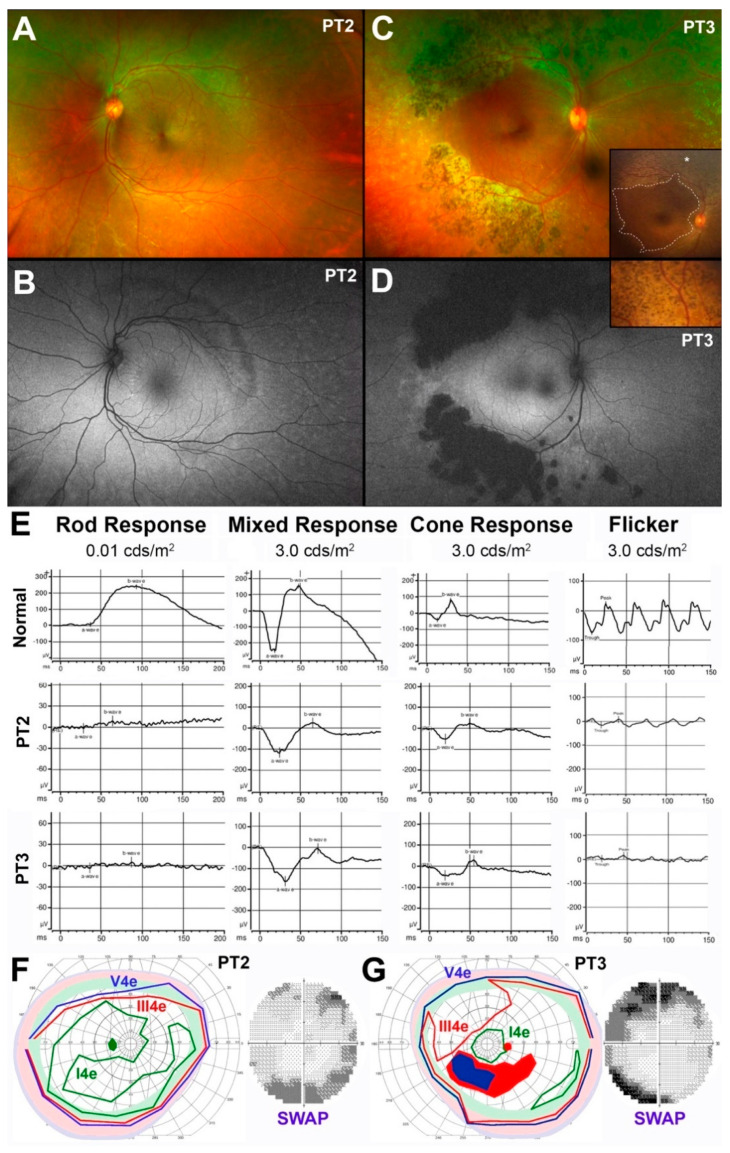
Clinical, functional, and imaging findings in pediatric patients 2 (PT2) and 3 (PT3). (**A**) Optos color fundus photograph from the left eye of PT2; (**B**) Optos fundus autofluorescence (FAF) image from the same eye of PT2, age 14yo; (**C**) Optos color fundus photograph from the right eye of PT3 at age 16yo. Insets: RetCam color images of PT3 obtained at age 6yo; (**D**) Optos FAF image from the same eye of PT3 at age 16yo; (**E**) Full-field flash electroretinogram (ffERG) responses of PT2 (14yo) and PT3 (16yo) compared to a normal example; (**F**) Semiautomated kinetic perimetry (SKP) and 30-2 blue-on-yellow (short-wavelength) automated perimetry (SWAP) from the left eye of PT2 (14yo); (**G**) SKP and blue-on-yellow 30-2 SWAP from the right eye of PT3 (16yo). The shaded pale green, pink and pale blue bands identify the normal typical limits for an SKP for each of the tested targets.

*Patient 2* and *3* (*PT2* and *PT3*). The clinical and functional presentations of PT2 and PT3 are illustrated in Figure 2.

Similar to PT1, PT2 (14 yo) did not exhibit any pigmentary deposits, and only whitish fleck-like nummular deep deposits outside the arcades were observed (Figure 2A). The extent of which was best appreciated on FAF imaging (Figure 2B), highlighting a band of hypo-AF at the supero-temporal arcade and disseminated faint speckled hyper-AF throughout most of the temporal periphery. There was also punctate and linear hyper-AF temporal to the disc inside the arcades. Unlike PT2, his 16 yo sister (PT3) presented with deep nummular retinal pigmentation at and outside the arcades associated with deep whitish fleck-like nummular deep deposits outside the arcades and disseminated punctate, exudate-like deposits most prominent in the nasal periphery (Figure 2C). The deep nummular pigment deposits in PT3 were present already at age 6 by RetCam retinal imaging (insets in Figure 2C,D), but were much smaller and far sparser in nature, while the area of central preservation had changed little over a 10-yr period (dashed line in inset in Figure 2C). FAF imaging highlights further the greater regional disease severity of PT3, revealing large confluent nummular patches of dense hypo-AF at the temporal arcades due to a combination of atrophic RPE and blockage from the nummular pigment deposits, and faint hyper-AF at and inside the arcades with both a diffuse and a speckled pattern (Figure 2D). Despite these clinical differences, the pattern of ffERG abnormalities of these two siblings was essentially identical and consistent with the diagnosis of ESCS (Figure 2E). Unlike PT1, both patients at this stage did not exhibit supernormal cone ERGs. Both PT2 and PT3 had partial relative ring scotomas corresponding to the areas of the fundus changes (Figure 2F,G). The lack of generalized supernormal S-cone-mediated function was confirmed in both cases by 30-2 chromatic automated perimetry (Figure 2F,G). Additionally, results in PT2 and PT3 showed a much better preservation of S-cone-mediated thresholds to blue-on-yellow 440 nm stimuli than to red-on-white 650 nm stimuli (latter not shown). No loci with supernormal S-cone-mediated thresholds were identified, and the central loci were within the normal range. Loci at the arcades showed abnormally reduced sensitivity by as much as −19 decibels (dB) in PT2 and −26 dB. This pattern resulted in mean defect across the field of approximately −4 dB for PT2 and −7 dB for PT3. This constellation of findings predicts that these two young patients met criteria for early Stage 3 disease, characterized by well (and preferentially) preserved, but not overtly supernormal, S-cone function, with some loci already exhibiting reduced S-cone-mediated function [39].

**Figure 3:**
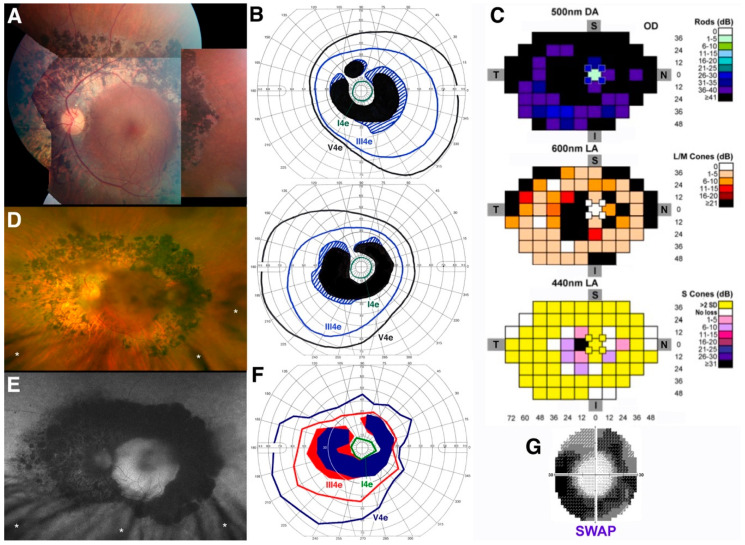
Clinical, functional, and imaging presentation in older adult patient 4 (PT4). (**A**) Color fundus photography composite from the left eye of PT4 at age 46; (**B**) Kinetic perimetry from both eyes at the same time point (top, right eye; bottom, left eye); (**C**) Full-field chromatic automated perimetry performed at age 46 in the left eye to dark-adapted (DA) rod-mediated 500 nm stimuli, 600 nm light-adapted (LA) L/M-cone mediated stimuli, and 440 nm LA stimuli on a bright yellow background (S-cone mediated—full-field short-wavelength automated perimetry (SWAP)); (**D**) Optos color fundus photo obtained from the same eye shown in (**A**) 11 years later (age 57); (**E**) Optos fundus autofluorescence (FAF) from the same eye at the same time point (note eyelash-induced artifacts in both (**D**,**E**), small white asterisks); (**F**) Semiautomated kinetic perimetry of the left eye at age 57; (**G**) Standard 30-2 SWAP performed at this same time point (age 57), 11 years after the full-field SWAP shown in (**C**).

*Patient 4* (*PT4*). The phenotypic expression of PT4, homozygote for the common R311Q *NR3E3* mutation, is summarized in Figure 3.

The management of his schitic macular complications [37] and, more recently, his secondary inflammatory autoimmune-mediated retinal and optic nerve manifestations have been previously reported [52]. At age 46, this patient presented with a ring of coalescent, mainly nummular deep retinal pigmentary deposits, accompanied by a band of RPE atrophy at the arcades and whitish punctate flecks in the mid- to far periphery and no vascular attenuation (Figure 3A). At this stage, overt subacute macular cystic changes could be observed clinically and by OCT mainly in the left eye (not shown here) and responded robustly to topical and systemic carbonic anhydrase inhibitors, with full acuity restitution [37]. Except for the macular cystic changes, all findings were symmetrical, and a partial absolute ring scotoma to kinetic perimetry under standard LA conditions characterized the visual field (Figure 3B). Full-field chromatic automated perimetry performed at this stage (Figure 3C) showed virtually no detection of DA rod-mediated 500 nm stimuli, except for at high stimulus intensity, where S-cone mediated detection is most likely. A partial absolute ring scotoma to 600 nm stimuli (L/M-cone mediated) presented on a standard white light-adapted background was seen, mirroring the one seen on kinetic perimetry. To 440 nm stimuli presented on a bright yellow background (full-field SWAP perimetry), S-cone mediated function was only mildly reduced in that same region of the partial ring scotoma, but was otherwise mostly supernormal (>2 standard deviation (SD) above the normal average) at other loci (yellow squares in Figure 3C). At this stage, this patient exhibited a phenotype meeting early Stage 3 classification, as seen in the much younger PT2 and PT3 [39].

The aforementioned secondary inflammatory autoimmune complications developed acutely in PT4 approximately 10 years later and were successfully managed [52]. Acuity at this stage was partially but transiently reduced by visually significant posterior subcapsular cataracts (still visible as a shadowing effect in both Figure 3D,E); however, near full acuity restitution that persists now at age 61 (see Table 1) was achieved after cataract surgery in both eyes. Similar to PT3, the fundus appearance of this patient one year after the inflammatory complications (at age 57) showed minimal clinical progression (Figure 3D), mainly reflected by denser and modestly more widespread nummular pigmentation and a more severe degree of RPE atrophy, illustrated further by FAF imaging (Figure 3E). Kinetic perimetry at this stage reflected these modest changes, with a mild increase in the size and severity of the partial ring scotoma and mild contraction of both the III4e and V4e isopters (Figure 3F). A standard 30-2 blue-on-yellow (SWAP) perimetry to 440 nm stimuli was obtained at this stage (inset in Figure 3G) and, while not permitting a one-to-one comparison with the same testing full-field procedure performed 11 years prior, direct results inspection showed definitely denser (yet still partial) ring scotomas at the arcades but fully normal thresholds centrally, indicating regional progression of S-cone disease severity at the arcades. The ensemble of these findings suggests mild disease progression to an early Stage 4 level [39], where deeper S-cone disease severity is starting to develop.

**Figure 4:**
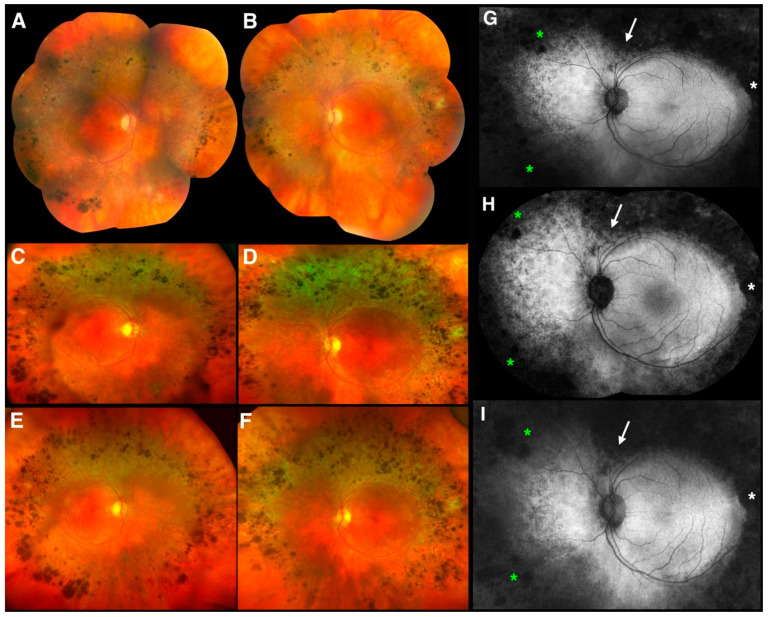
Clinical and imaging findings in an older adult patient 5 (PT5). Color photography composite images from each eye of PT5 at age 59 ((**A**) right eye; (**B**) left eye), Optos color photographs from each eye of PT5 at age 66 ((**C**) right eye; (**D**) left eye) and at age 69 ((**E**) right eye; (**F**) left eye). Fundus autofluorescence (FAF) images from the left eye of PT5 at corresponding time points ((**G**) Optos FAF image at age 59; (**H**) Spectralis FAF composite image at age 66; (**I**) Optos FAF image at age 69). The white arrow, white asterisk, and green asterisks point to areas of increasing hypo-autofluorescence over the years (see text for details).

**Figure 5:**
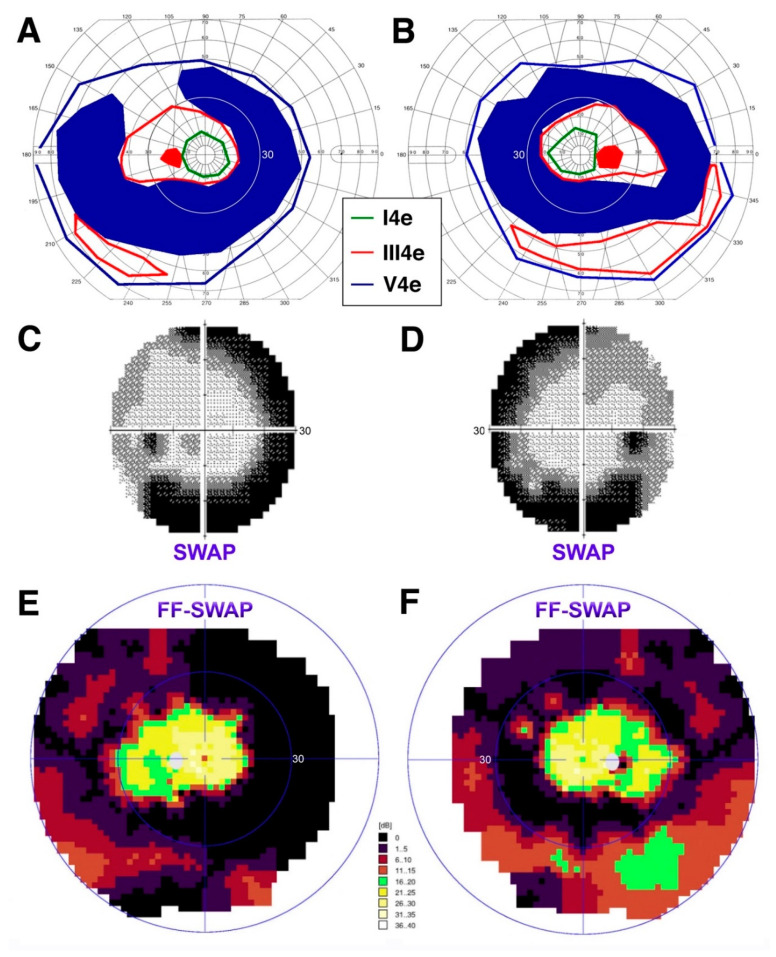
Perimetric findings in an older adult patient, PT5. Semiautomated kinetic perimetry (SKP) from each eye at age 68 ((**A**) left eye; (**B**) right eye). Standard 30-2 short-wavelength automated perimetry (SWAP) findings at the same time point ((**C**) left eye; (**D**) right eye). Full-field (FF)-SWAP findings at age 69 ((**E**) left eye; (**F**) right eye). To facilitate landmark recognition, the inner thin blue ring on the color FF-SWAP plots marks the 30-deg limits (same as the standard 30-2 SWAP and as the thin white circle on the SKP plot).

*Patient 5* (*PT5*). Lastly, the clinical and functional phenotypes exhibited by PT5 are illustrated in Figure 4 and Figure 5, respectively.

Fundus exams over a 10-year period showed the most widespread of the deep nummular pigmentary changes at the arcades seen in any of our patients with ESCS, associated with deep retinal greyish discoloration and a progressive accumulation at the arcades over the years (Figure 4). At baseline (age 59), the pigmentary changes were sparser and peripheral to the arcades, where the greyish discoloration prevails (Figure 4A,B). Seven years later (age 66), both the pigmentation and the greyish discoloration become denser and more noticeable, having grown mildly centrifugally and respecting the vascular arcades (Figure 4C,D). At age 69, after 10 years of follow up, both pigmentation and greyish discoloration have further increased in the same band outside the vascular arcades (Figure 4E,F). There is, however, always persistent excellent posterior pole preservation, documented over a 10-year span, despite overt suffering of the intact retinal tissue, as suggested by the marked and diffuse hyper-AF on WF-FAF across the central regions spared by the pigmentary reaction (Figure 4G,I). The latter is reflected also by the visual acuity findings (Table 1), which was not affected by any cystic macular changes at this stage (macular OCT not shown). There was mild centripetal increase in the hypo-AF defects of the over the 10-year period, with an increasing superior V-shaped notch above the disc (white arrows), a small peripheral notch on the temporal side (white asterisks), and increasing peripheral hypo-AF due to increased nummular pigmentation (green asterisks).

The ffERG (not shown) was similar to that of PT2 and PT3, configuring a typical ESCS phenotype, except that responses were scaled down in size, simply consistent with more advanced disease severity. Three and a half years later, all ffERG responses were unchanged except for a minor reduction in the mixed a-wave amplitude which was within the test-retest variability range (not shown). Thus, per se, the ffERG was important in the diagnostic process of establishing a diagnosis of ESCS, but not particularly informative in the longitudinal follow-up assessment over this period of time.

The greater disease severity at the arcades is further attested to by the perimetric findings, which reveal large absolute ring scotomas at age 68 (Figure 5A,B). S-cone sensitivity was measured one year apart with two methods. Standard Humphrey SWAP 30-2 perimetry shows good, but mildly reduced, central S-cone mediated function and greater regional S-cone disease severity at the arcades (Figure 5C,D), corresponding perfectly to the absolute ring scotomas seen on kinetic perimetry. Subsequent supplemental full-field SWAP perimetry (utilizing the Octopus 900) confirmed these findings and provided evidence for persistent albeit reduced S-cone mediated sensitivities also peripherally, corresponding to the areas of best preservation of the III4e target on kinetic perimetry (Figure 5E,F), which were unchanged at age 69 compared to what shown in Figure 5A,B. Taken together, this patient appears to exhibit a phenotype most consistent with that of a late Stage 4 level [39], where deeper yet S-cone disease severity is occurring, but with good retention of central sensitivity and persistent but reduced peripheral S-cone mediated function.

### 3.2. Kinetic Visual Field Decay over Time in ESCS

**Figure 6:**
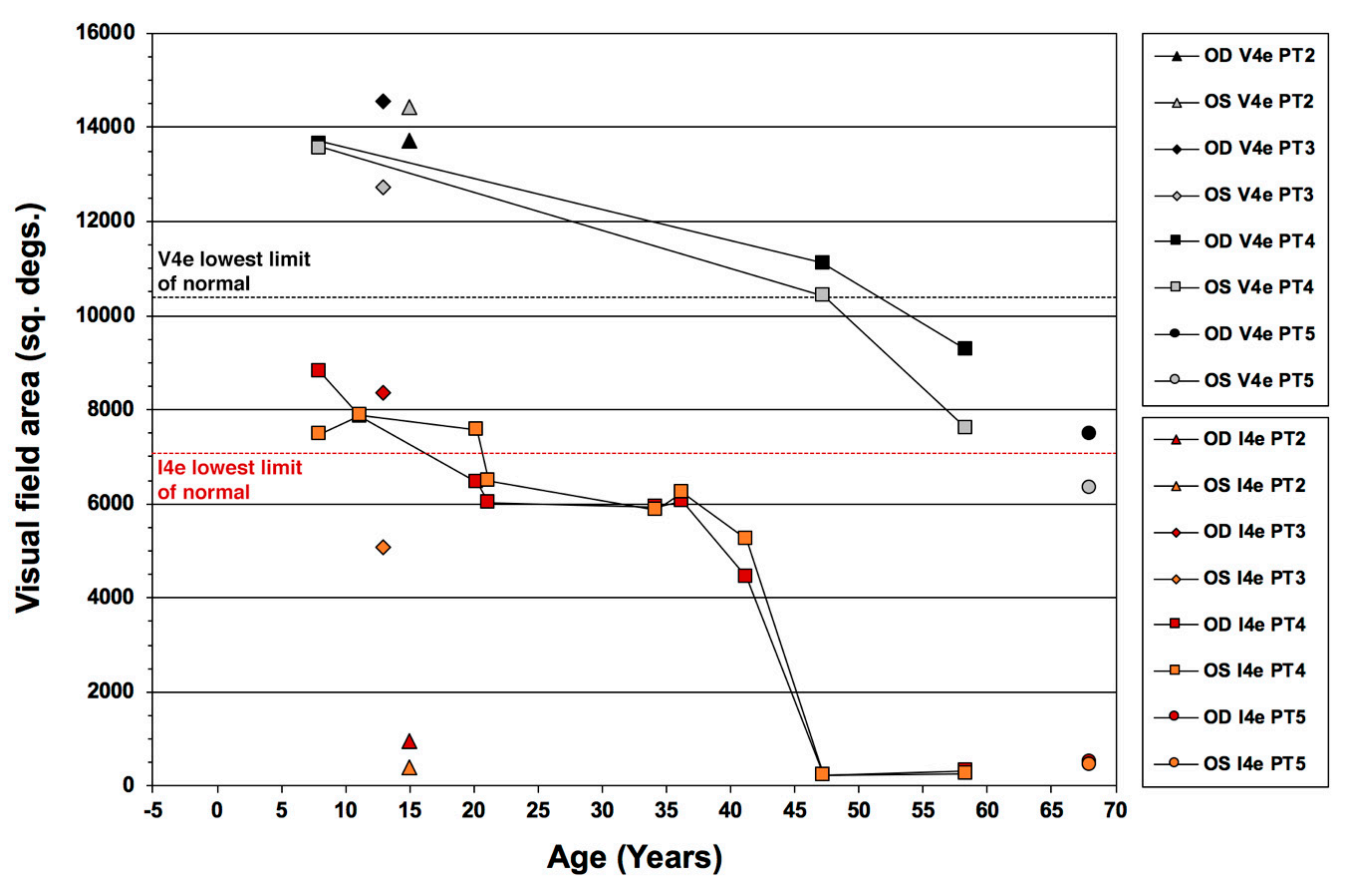
Pattern of kinetic visual field decay over time in patients with the Enhanced S-Cone Syndrome (ESCS). Areas for the V4e and I4e test targets are defined in square degrees (sq. degs.) for the right eye (OD) and left eye (OS) of patients 2 (PT2) through 5 (PT5).

Figure 6 illustrates the kinetic visual field areas for the V4e and I4e isopters as a function of age in our case series, and features data over 5 decades for PT4, allowing us also to gain insight on the long-term disease progression over time both for this particular patient and for ESCS in general.

High quality kinetic perimetry results were also available since his childhood, starting at age 8 yo when the patient was first diagnosed as affected (erroneously thought to be retinitis pigmentosa). Plots of the V4e and I4e areas over time show that the I4e area was still within the normal range during the first 10 years since diagnosis (age 18), followed by a mild decline below the normal limits during the subsequent 2.5 decades of life and then a sharper decline in the I4e area after the 30th year of follow-up from baseline (early 40’s), declining to the present size (approx. 3.5% of the lowest limit of the normal range) at age 46 and essentially maintained for the subsequent 11 years (through age 57). During this period of time, the overall size of the GVF to the V4e target remained within the normal limits for the first 4 decades of follow up (age 46), followed by mild yet measurable absolute peripheral loss by age 57. With the exception of the I4e area data for PT2, the outlier in this case series (red- and orange-filled triangle symbols) and the youngest for which reliable perimetries could be performed, all other data points fit well the longitudinal behavior of the visual field of PT4.

Overall, these data indicate a relatively slow disease progression with good preservation of desensitized peripheral vision outside the ring scotomas and retention the central 10–15 degrees of central visual field with excellent central retinal function well into the 7th decade of life. Our findings mirror closely the behavior of the V4e target area for kinetic visual field decay reported in ESCS by Garafalo et al. [38] and add information about the I4e target, which appears to be far more likely to experience significant decline in size, especially around the 4th to 5th decade of life for affected patients. Since the V4e target is more indicative of vision quantity, whereas the I4e target is more so of its quality, this pattern is consistent with a greater qualitive than quantitative decline in vision over time in ESCS. This behavior is reminiscent of what we previously observed in Usher syndrome type 2 [46]. While the latter condition is far more aggressive and progresses with different kinetics, also in Usher syndrome type 2 there is a lag between the decay of the V4e target compared to the I4e target, consistent with quality of vision declining ahead of quantity.

### 3.3. Longitudinal Study of rd7 Retinal Degeneration Shows a Slow Progression of Disease

**Figure 7:**
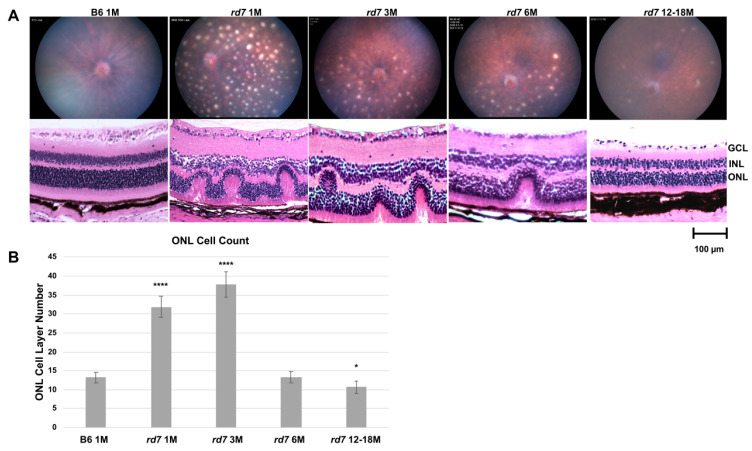
Fundus and histology of *rd7* mice shows progressive retinal degeneration. (**A**) Fundus and histology staining analysis of *rd7* mice track the progression of retinal degeneration compared to B6 control mouse model. Four time points: 1, 3, 6, and 12–18 months were evaluated. (**B**) ONL cell counts in 1 and 3 month *rd7* animals show an increase of ONL apparent in whorls compared to the B6 control (**** *p* ≤ 0.0001 and *p* ≤ 0.0001, respectively), followed by a sharp degeneration by 6 months. ONL cell counts in 12–18 month animals show a statistically significant decrease (* *p* ≤ 0.05). Standard error (SE) bars indicated for each time point. N = 5.

Fundus imaging of adult *rd7* mice was collected at 1,3,6, and 12–18 months to evaluate retinal degeneration disease onset rate and characterization. As previously published, retinal dysplasia is characterized by whorls and rosettes apparent at postnatal day 10 (P10) and retinal spots observable by fundus imaging at eye opening (P14) [24,42,53], the time at which has been previously indicated as the early stage of disease [24]. 1-month *rd7* mice exhibit robust pan-retinal spotting (Figure 7A) that fade as the disease progresses with concomitant loss of photoreceptors as the animals age.

As previously reported, the pan retinal spots appear to correlate with whorls and rosettes, shown at 1 month by hematoxylin and eosin (H/E) staining (Figure 7A). The rosettes as well as the retinal spots resolve with degeneration as the disease progresses. Previously published data indicate that these whorls and rosettes are due to an increase in blue opsin-expressing cone photoreceptor cells [24]. In order to carefully evaluate *rd7* photoreceptor loss over time, vertical rows of nuclei in the ONL were counted across the central retina. It is important to note that within a retinal section depending on the region of the whorl in that section, ONL counts can vary significantly. Counts were taken from vertical column of nuclei within the whorls, spanning approximately 100 µ of retina. The initial over proliferation shows an increase of ONL thickness well above the normal levels at 1 and 3 months when whorls and rosettes are most abundantly present, followed by a sharp decline by 6 months with continuing degeneration at 12–18 months when ONL thickness is reduced to below normal levels (Figure 7B).

**Figure 8:**
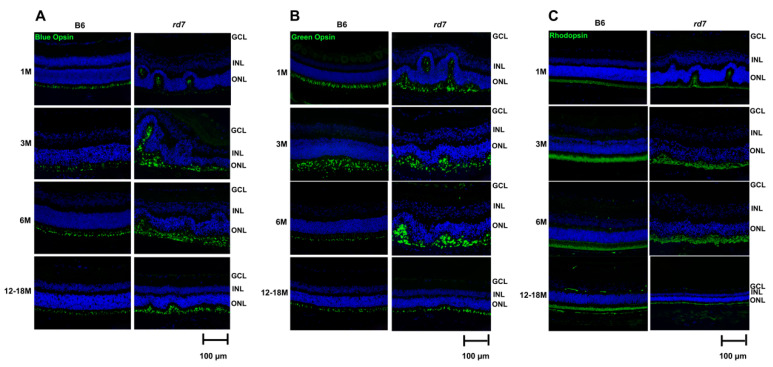
*rd7* mouse model presents abnormal *Cone* and Rod function. Immunohistochemistry (green) staining of blue opsin (**A**), green opsin (**B**), and rhodopsin (**C**) as well as 4,6-Diamidino-2-Phenylindole, Dihydrochloride (DAPI, blue) show abnormal retinal structure in *rd7* mice as compared to the B6 control at 1, 3, 6, and 12–18 months. N = 5.

Our prior studies showed *rd7* mice have an increase of blue opsin-expressing photoreceptor cells that disrupt retinal topography. Here we examined whether green cones and rod photoreceptors are also impacted by the disruption of retinal topography. Interestingly, immunohistochemistry (IHC) labeling of cone and rod photoreceptors show both rods and cones in the whorls and rosettes indicating that, in mice, disruption of retinal topography impacts all *rd7* photoreceptor cells (Figure 8).

The whorls and rosettes resolve as retinal degeneration progresses in *rd7* mice with concomitant loss of rod and cone photoreceptor cells.

**Figure 9:**
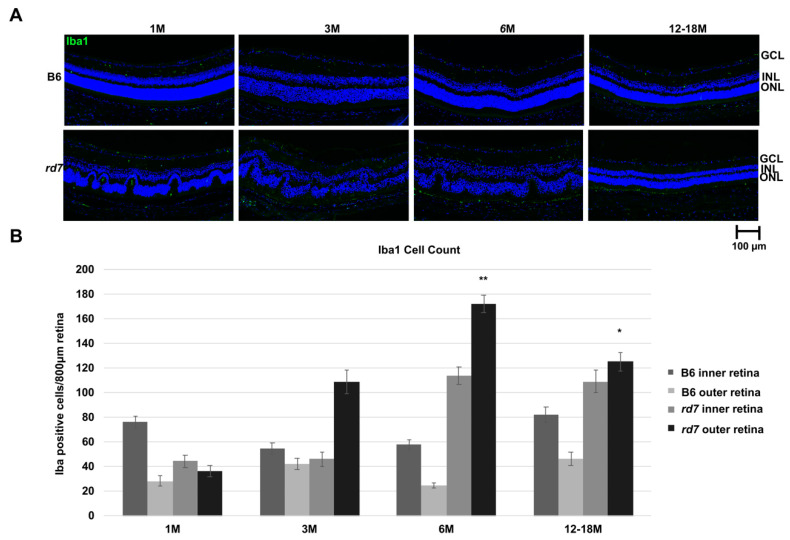
Ionized calcium binding adaptor molecule 1 (Iba1) labeling indicates presence of increased inflammation in *rd7* mice as disease progresses. (**A**) Immunohistochemistry labeling of Iba1 (green) and 4,6-Diamidino-2-Phenylindole, Dihydrochloride (DAPI, blue) show increased Iba1 in *rd7* mice at 3, 6, and 12–18 months (M) compared to the B6 control mice (GCL = ganglion cell layer; INL = inner nuclear layer; ONL = outer nuclear layer); (**B**) Iba1 cell counts of inner and outer retina of *rd7* mice show increased Iba1 in outer retina at 3, 6, and 12–18 months. Both 6 month and 12–18 months showed a statistically significant increase in Iba1 cell count for the outer retina for *rd7* mice compared to the B6 control. Inner retina (GCL, INL); Outer retina (ONL). *p* values indicated by asterisks (*p* ≤ 0.01 and *p* ≤ 0.05, respectively). Standard error (SE) bars indicated for each time point. N = 5.

A recent study found that macrophage infiltration may be associated with the whorls and rosettes in *rd7* [54]. We evaluated the ionized calcium binding adaptor molecule 1 (Iba1) to determine if microglia or macrophage are found in the whorls of *rd7* retinas, and if their presence changes as the disease progresses. Iba1 is an actin-binding protein that enhances membrane ruffling and RAC activation, and it is thought to play a role in microglia activation and function [55,56]. It is suggested that activated microglia expressing Iba1, which are present at sites of injury, are responsible for cell migration and phagocytic activity to further damage viable tissue [55,56,57,58]. Interestingly, IHC labeling shows the presence of Iba1 in *rd7* mice throughout *Nr2e3* disease progression in *rd7* compared to the B6 control mice (Figure 9).

Iba1 expressing cells in the normal (B6) retina remain relatively constant throughout the lifespan of the mouse and appear to be more in the inner versus outer retina. In contrast, Iba1 expressing cells in *rd7* retina are similar to normal levels at 1 month, however by 3 months while the inner retina is similar to B6, the outer retina, where the *rd7* disease manifests, shows significantly higher levels of Iba1. As retinal disease progresses by 6 months, Iba1 cells continue to increase in the outer retina and into the inner retina as well and remain more than normal at 12–18 months. Expression of Iba1 is thus coincident with the degeneration of photoreceptor cells.

**Figure 10:**
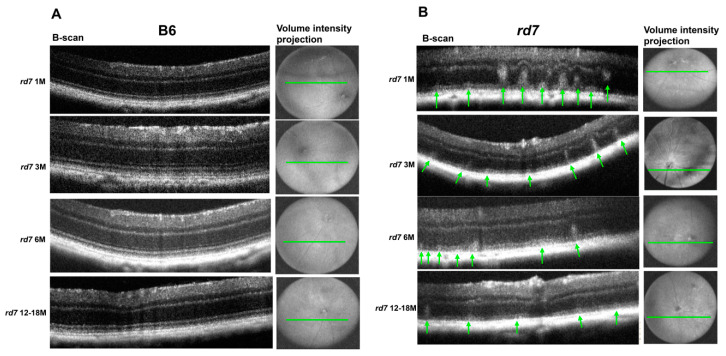
Whorls and rosettes associated with *rd7* retinal degeneration are perceptible with Envisu optical coherence tomography (OCT) imaging. (**A**) B6 control and (**B**) *rd7* OCT images. Whorls and rosettes apparent in *rd7* images (green arrow). Four time points were evaluated: 1, 3, 6, and 12–18 months. N = 5.

Longitudinal OCT imaging analysis shows a similar pattern to that of histology findings, illustrating a disruption of retinal topography and integrity by whorls and rosettes that diminish over time (Figure 10).

OCT imaging can detect the whorls and rosettes that were observed in histology sections. Furthermore, consistent with histological analysis, OCT imaging shows that the most numerous whorls and rosettes are apparent at 1 and 3 months of age. OCT images collected from 6-month old *rd7* mice show a significant decrease in number and size of whorls and rosettes that continues to decline as the disease progresses in 12 to 18 month *rd7* animals. Furthermore, OCT analysis shows progressive reduction in retinal thickness compared to the B6 wildtype mice (Figure 10). These results underline the slow progression of *Nr2e3*-associated retinal degeneration in *rd7* and illustrate the value of in vivo imaging as a way to assess *NR2E3* disease progression in patients.

### 3.4. Retinal SD-OCT Imaging Findings in ESCS Patients Mirror Findings in Rd7 Mice

**Figure 11:**
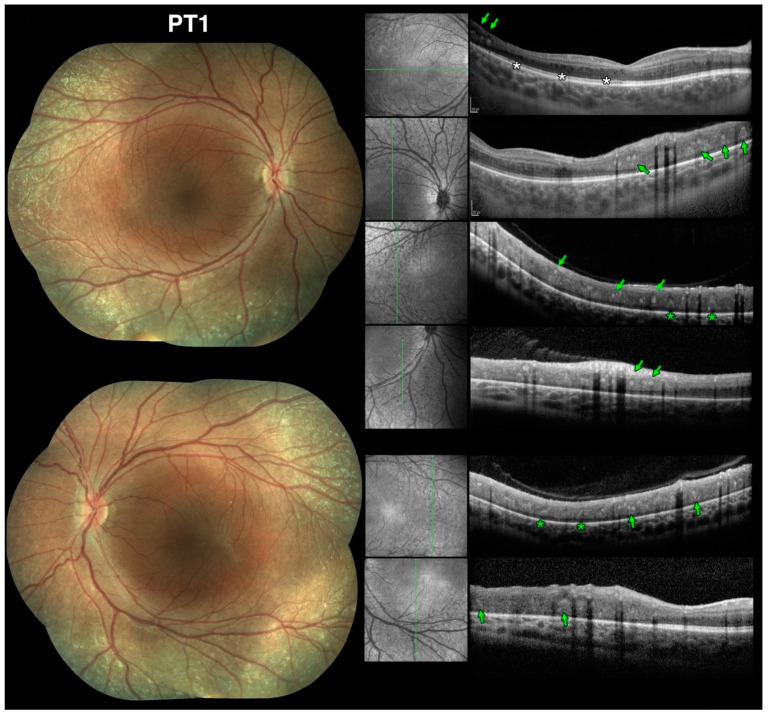
Spectral domain optical coherence tomography (SD-OCT) findings at the vascular arcades in early stage Enhanced S-Cone Syndrome (ESCS) patients mirror findings in *rd7* mice. Color fundus photography composites from Stage-1 patient 1 (PT1) at the age of 9yo (**left**) and SD-OCT scans (**right**) with accompanying red-free scanning laser ophthalmoscope images to permit identification of the scanned location vis-à-vis the color images. White asterisks indicate microcystic changes. The colored markers identify 2 of the 5 types of lesions we characterized. In this part of the figure: (1) green arrows identify well defined, discrete, nummular, intraretinal hyperreflective foci (irHRFs) yielding moderate hyperreflectivity in the intermediate retinal layers; (2) green asterisks identify fainter cone-shaped irHRFs that appeared to stem from the RPE and that were seen protruding through the outer retinal third. See text for complete discussion.

**Figure 12:**
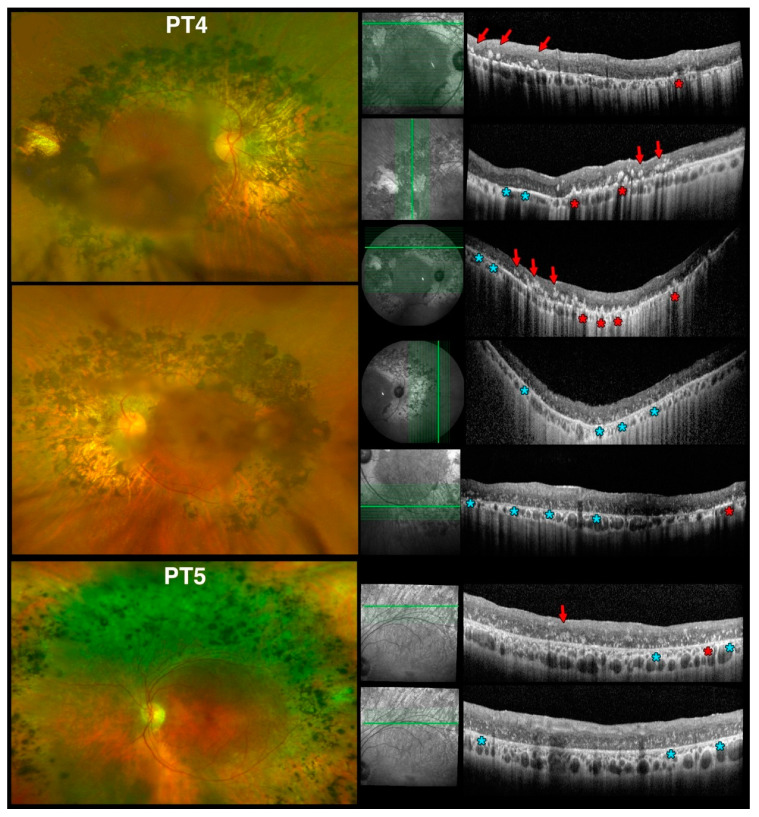
Spectral domain optical coherence tomography (SD-OCT) findings at the vascular arcades in older patients with Enhanced S-Cone Syndrome (ESCS) patients mirror findings in *rd7* mice. Optos color fundus photos from early Stage-3 patient 4 (PT4), age 61 yo, (**top left**) and late Stage-4 patient 5 (PT5), age 69yo, (**bottom left**) and SD-OCT scans (**right**) with accompanying red-free scanning laser ophthalmoscope images to permit identification of the scanned location vis-à-vis the color images as in (Figure 11). The colored markers identify 3 of the 5 types of lesions we characterized. In this part of the figure: (1) red arrows point to brighter, well-defined nummular intraretinal hyperreflective foci (irHRFs) seen intraretinally; (2) red asterisks identify similarly much brighter, cone-shaped irHRFs originating from the RPE level; (3) light blue asterisks identify fainter, punctate, disseminated irHRFs in the outer retinal third. See text for complete discussion.

Previous studies note that the majority of the meaningful changes in retinal function in ESCS, especially with regard to S-cones, occurs at and just outside the vascular arcades, roughly between 10 and 40-degrees of eccentricity from the fovea [38]. This was certainly verified to be the case in our patients harboring *NR2E3* mutations and presenting with an ESCS phenotype. Additionally, ESCS patients tend to exhibit deep nummular changes at and outside the same pericentral location as one of the most recurrent disease features [38]. This location is where most of the visual field loss and S-cone function changes tend to occur [27,28,35,37,59,60]. These characteristics were observed in our patients as well and was noticeable before pigmentary changes occurred. Thus, we sought to investigate further the nature of these nummular changes by obtaining SD-OCT scans performed at or near the arcades, examples of which are illustrated in Figure 11 (Stage 1 patient) and Figure 12 (Stage 3 and 4 patients).

**Figure 13:**
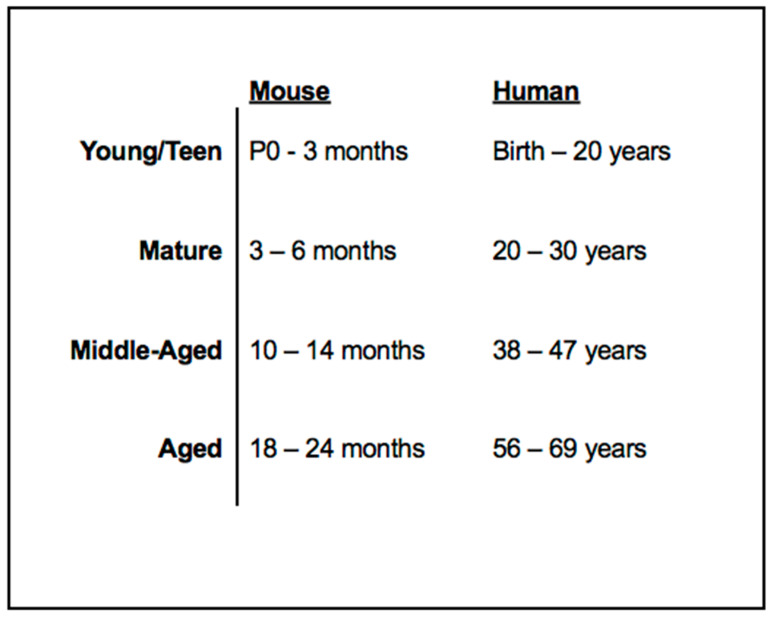
Mouse and human life phase equivalents. C57Bl/6J mice maturation rate and life phases were correlated to that of a healthy human individual. Data adapted from Flurkey et al., 2007 [45].

Here, we show that the unique retinal nummular intraretinal hyperreflective foci (irHRFs) seen on OCT imaging in *rd7* mice, which are known to correspond to intraretinal deep photoreceptor cell rosettes and whorls, can be seen on SD-OCT scans in ESCS patients, where nummular deep changes are seen since the earliest disease stages, and persist—and evolve—in later disease stages. Five main types and characteristics of the irHRFs were identified, which characterized different disease stages. Figure 11 shows OCT findings in PT1, the youngest in our case series, exhibiting a phenotype meeting Stage 1 criteria [39]. Age-wise, this 9-yo patient would correspond to 1- to 2-mo *rd7* mice (see Figure 13 for age approximations between species).

This age corresponds to when photoreceptor rosettes and whorls are most prominent in the *rd7* mouse. This patient, who had no pigmentary changes at the time of evaluation, presented with main two types of well defined, discrete, nummular, moderately hyperreflective irHRFs seen in the intermediate retinal layers (green arrows) and fainter cone-shaped irHRFs that appeared to stem from the RPE and that were seen protruding through the outer retinal third (green asterisks). We interpret these OCT changes as corresponding to the photoreceptor rosettes and whorls, respectively, seen on *rd7* mouse histology and confirmed by OCT imaging. Of note, on the red-free SLO images that accompany SD-OCT imaging, punctate nummular changes that correspond to these OCT changes are evident even when there are no ophthalmoscopically visible anomalies. This patient also exhibited mild microcystic changes in the macular region (white asterisks), as often seen in ESCS.

OCTs obtained in our older patients (PT4 and PT5) meeting criteria for early and late Stage 4 level [39], respectively, are shown in Figure 12. Age-wise, these patients (61 and 69 years of age, respectively) would correspond to 18- to 24-mo mice. Clinically, both PT4 and PT5 had confluent nummular deep pigment deposits at the arcades. They too exhibited irHRFs, but at these later disease stages they differed in appearance. In PT4, much brighter, well-defined nummular irHRFs were seen (red arrows), associated with similarly much brighter, cone-shaped irHRFs originating from the RPE level (red asterisks). The brighter, well-defined nummular irHRFs corresponded to areas of deep nummular pigmentation seen clinically. We interpret these as degenerative progression of the same lesions seen in PT1, thereby corresponding to degenerating photoreceptor rosettes and whorls, respectively. PT4 also exhibited fainter, punctate, disseminated irHRFs in the outer retinal third (light blue asterisks). The latter features were present in areas devoid of pigmentary deposits and were seen where, by perimetric criteria, there was well preserved retinal function.

In areas of deep nummular pigmentation outside the arcades, OCTs in PT5 were comparable to the first three OCT scans from PT4. The fainter, punctate, disseminated irHRFs in the outer retinal third (light blue asterisks) dominated retinal microanatomy at the transition between the superior band of retinal pigmentation and RPE atrophy and healthier retina (bottom panels in Figure 12). Scans at the inferotemporal arcade in PT5 (Figure S2A,B), which was the least affected and corresponded to areas not exhibiting scotomas on perimetry, also presented with fainter, punctate, disseminated irHRFs in the outer retinal third dominated (light blue asterisk type lesions) with some occasional better defined nummular lesions (green asterisk type lesions, as seen in PT1) and rare far peripheral nummular irHRFs (red arrow type lesions). The late fading of whorls and rosettes in *rd7* mice is associated with photoreceptor loss. Thus, due to the association with better visual function preservation on perimetry, we suggest that the fainter, punctate, disseminated irHRF changes seen in ESCS patients (light blue asterisks in Figure 12) may represent interval areas between degenerated photoreceptor rosettes and whorls, as seen in later stages of *rd7* mice. Alternatively, they may represent persistent but fading remnants of rosettes and whorls that have not undergone degenerative events, which are typically expressed in ESCS patients by subretinal or deep intraretinal pigmentary changes (i.e., the ones that appear as bright, well-defined nummular or cone-shaped irHRFs originating from the RPE level on SD-OCT imaging—red asterisks and red arrows, respectively, in Figure 12).

In the 2 older children (PT2 and PT3, not shown) who had a mix of the same ophthalmoscopic characteristics of PT1 with nummular deep pigment deposits, meeting criteria for early Stage 3 disease [39], the irHRF changes were at an intermediate stage between PT1 and PT4, with an admixture of the discrete, well defined lesions but none of the fainter, punctate, disseminated irHRFs as seen in the outer retinal third of PT4 and PT5 were found in either PT2 and PT3. Thus, the latter features appear to be unique to later disease stages, so long as these irHRFs do not coincide with pigmented lesions (i.e., the aforementioned red arrow or asterisk-type lesions in Figure 12). OCT changes also appear to be associated with better visual function than neighboring areas and would predict areas still suitable for therapeutic intervention. It is important to note how these irHRFs are not normally observed in the central macular region of ESCS patients, which is where SD-OCT scan are normally performed in a standard clinical setting (compare Figure S2C). Thus, they can be almost exclusively appreciated via custom scans at the arcades or nasal to the disc in areas exhibiting the nummular changes seen ophthalmoscopically, or via wide-field SD-OCT (as shown in Figure S2).

### 3.5. Autofluorescence-OCT Macular Imaging Correlates in ESCS

It has been previously reported that there may be a direct correspondence between intraretinal putative rosettes as seen on macular SD-OCT scans in ESCS patients and macular hyper-AF spots on FAF imaging [54]. We did not observe rosette and fold-like whorls in the macular region in any of our patients, nor any such correspondence between the rosette and fold-like whorls seen at the vascular arcades and hyper-AF foci. We also did not observe nummular hyper-AF foci in the macular region in any of our patients. Thus, we were unable to confirm this specific observation. This may in part be due to the fact that the patient with those particular features reported by Wang et al. was 6 yo, thus that presentation may represent a finding unique to earlier disease stages [54].

The only patient who exhibited a few, mostly linear, hyper-AF changes in the macular region around the fovea was PT2 (Figure S3). To determine if these hyper-AF changes corresponded to retinal changes reminiscent of what reported by Wang et al. [54], we used a multi-modal approach, overlaying SD-OCT macular scans with the FAF image. The following two findings emerged in PT2: (a) as reported both in ESCS patients and in the *rd7* mouse, there were wavy abnormalities at the level of the ONL, OPL, and INL (Figure S3A, white arrows)—these changes were not observed centrally in PT2, or in any other patient in our series; (b) the hyper-AF changes were associated with different, unique SD-OCT changes: a macular FAF image of PT2 is shown in Supplemental Figure S3B, and the location of 7 SD-OCT scans that exhibited intraretinal changes reminiscent of what we observed exclusively at the arcades is presented (green lines, shown interrupted so as not to block the hyper-AF changes seen on FAF imaging)—the detail of these 6 SD-OCT scan is presented in Supplemental Figure S3C. Except for a single cone-shaped faintly hyperreflective change seen on scan 1 (white arrow), no other discrete, nummular or cone-shaped *hyper*-reflective change as seen at the arcades in this and all other patients was observed. Instead, in PT2, hyper-AF at the level of the RPE appeared to coincide mostly, if not exclusively, with intraretinal *hypo*-reflective spots and cleft-like changes, typically abridging the ONL, OPL, and INL (white arrows). Unlike the case reported by Wang et al. [54], no overt underlying hyperreflective change at the level of the RPE level was seen in PT2 corresponding to the faint, linear hyper-AF changes seen on FAF.

## 4. Discussion

Our study utilized an in-depth analysis of functional and imaging findings of ESCS patients at various disease stages to draw a parallel between *NR2E3*-related disease in ESCS patients and the *rd7* mouse. These findings illustrate a correlation of phenotypes and confirm the value of *rd7* mice as a model of *NR2E3-*associated retinal disease. Autosomal recessive *NR2E3*-related disease manifests as a slow disease progression in ESCS patients, with good visual acuity preservation in the vast majority of cases, good overall macular function preservation, good retention of far peripheral vision, and relatively slow progression of kinetic visual field loss as noted by previous studies [22,31,32,60,61], which is consistent with what we observed in our patients. A retrospective literature analysis on the clinical characteristics of the Nr2e3 patients shows a gradual decline of the visual acuity and reduced ERG over time (Table S1) [24,27,28,35,38,41,59,60,62,63,64,65,66,67,68,69,70,71,72,73,74,75,76,77,78,79,80,81,82,83,84,85,86,87,88,89,90,91]. This pattern of progression is similar to the observations of *rd7* mice.

### 4.1. Abnormal Retinal Topography Due to over Proliferation of Cone Cells in Rd7 Mice

These studies expand on our prior discoveries on the pathology of *rd7* mice reported in previous studies [24]. Interestingly, while retinal topography is disrupted due to over proliferation of cone photoreceptor cells, the distribution of rods and cones across the *rd7* retina remains relatively normal. Additionally, as in the normal mouse retina, blue opsin dorsal-ventral gradient is observed and there is uniform, pan retinal expression of green opsin and rhodopsin. This study revealed that *rd7* mice have an increase in both blue and green cone cells. It is likely that, as in the normal mouse retina, *rd7* retinas have co-expression of blue and green opsin in their cones. In the mouse, as opposed to humans that express single opsin genes in each photoreceptor cell, most cone photoreceptor cells express both blue and green opsin, and that remains true for *rd7* mice. On the other hand, rod cells do not appear to show an increase in number; however, labeling of retinal morphology illustrates rod cells under and within whorls and rosettes, suggesting their structure and topography are disrupted as well. As *Nr2e3* disease progresses, there is progressive degeneration of both rods and cones that results in resolution of the whorls and rosettes. Additionally, retinal spots in the *rd7* mouse fundus fade with disease progression. This photoreceptor cell loss allows for a decrease in number of cone nuclei under and within the disrupted ONL, potentially allowing for this attenuation of the whorls and rosettes as confirmed by retinal morphology during the later stage of disease.

### 4.2. Correspondence between SD-OCT and Autofluorescence Imaging Findings in Patients with ESCS

We were able to unambiguously document the correspondence between the photoreceptor rosettes and fold-like whorls seen histologically in *rd7* mice and changes seen at the vascular arcades in ESCS patients by SD-OCT criteria. Additionally, differential SD-OCT characteristics were identified between retinal microanatomical features associated with better preserved retinal function as assessed by perimetric sensitivity. Furthermore, as the disease progresses, lack of discrete irHRFs and presence of fainter, more granular irHRFs on SD-OCT was found in areas that were less affected both clinically and functionally. We were, however, unable to replicate the finding reported by Wang et al. [54] in a younger patient of correspondence between rosette-like structures on macular OCT and hyper-AF spots on FAF imaging. Rather, we found that, in the only patient who exhibited macular hyper-AF spots (PT2), these changes corresponded best to *hypo*-reflective intraretinal clefts and spots. The exact meaning of this particular finding is presently uncertain and deserves further investigation. We also suggest that the discrepancy between our imaging correlates and those identified by Wang et al. [54] may be due to the younger age of the patient in which it was reported and may, thus, represent an early and perhaps transient characteristic of ESCS patients. Our findings in PT2 (14 yo) may also represent a subsequent transient phase of the disease. Additional studies in larger and younger case series will be necessary to confirm this possibility.

### 4.3. Correlations between In Vivo Imaging across Species, Retinal Function in Humans, and Histopathologic Findings in the Rd7 Mouse Suggest a Role for Immunocompetent Cells in Disease Pathophysiology in NR2E3-Related Disease

Wang et al. showed that *rd7* mice exhibit colocalization of hyper-AF near the RPE at the base of the fold-like whorls with the macrophage marker F4/80 [54]. It has been subsequently suggested from studies of *Nrl*^−/−^ mice that a primary anomaly of the photoreceptor outer segments in ESCS impairs RPE outer segment phagocytosis [92]. We observed a lack of correspondence between hyper-AF lesions on FAF imaging and the hyperreflective intraretinal rosette-like or fold-like cone-shaped lesions—rather, these changes corresponded to intraretinal macular *hypo*-reflective spots and cleft-like changes. This suggests that, at least in the macular region, the hyper-AF changes seen on FAF may reflect foci of visible accumulation of hyper-AF material in the RPE due to the noted defective RPE outer segment phagocytosis impairment. This could be potentially associated with local macrophage infiltration at—or resident retinal microglia migration to—the outer segment/RPE interface, as it has been shown to occur in immune restoration disease (IRD) models [93]. This has indeed been observed also in *rd7* mice [94].

Iba1 labeling of *rd7* retinas in our current experiments confirms Iba1 expression primarily in the outer retina, at the base of the fold-like whorls, and milder, more discrete Iba1 expression elsewhere in the retina in *early* disease stages (1–3 months). This is followed by disappearance of Iba1 reactivity at the base of the whorls observed at 3 and 6 months, but a more abundant punctate expression above and in between where the residual whorls persist. Interestingly, this pattern closely resembles closely the punctate hyper-reflective lesions seen on SD-OCTs from patients with ESCS that appear to be associated with better vision preservation. Iba1 expression continues to increase with disease progression in the *rd7* retina, peaking at 6 months, then decreases at 12–18 months of age. It is not known if these patterns of Iba1 expression represent the exact equivalent of the human SD-OCT findings and, thus, if macrophage infiltration and/or intraretinal microglial cell migration may be taking place at these locations in ESCS patients. Since it has been suggested that microglial cell migration may serve, at least at some disease stages, a beneficial role [93], their possible association with retinal areas with better preserved S-cone mediated vision appears plausible. It is also not clear if these immunocompetent cells, or declining numbers thereof, may also be at play in later disease stages and perhaps explain, at least in part, the susceptibility to inflammatory manifestations exhibited late in the course of disease by patients like PT4. We are aware of at least one other case of molecularly confirmed ESCS with late inflammatory complications that, like PT4, responded well to steroids and systemic IMT (John R. Heckenlively, 2016, personal communication). Inflammatory complications in IRDs are common and an increasingly emerging side of this group of disorders for both pathophysiologic characterization and therapeutic purposes [52]. Thus, while inflammatory complications may occur also in ESCS, and their reason may not yet be completely understood, it is reassuring that they can be readily identified and, perhaps more importantly, successfully treated in affected patients. It will likely prove interesting and insightful to further investigate the role that macrophages and resident microglial cells may be playing in the pathogenesis of *NR2E3*-related disease in future studies, and the *rd7* mouse appears to exhibit optimal features to investigate further this particular facet of the condition.

### 4.4. The Value of In Vivo Retinal Imaging in NR2E3-Related Disease: Parallels, Differences, and Further Insights into Disease Dynamics

SD-OCT has been previously described as a powerful tool to diagnose several retinal degenerative as well as neurodegenerative diseases [95,96,97,98,99], In addition to changes at the vascular arcades in humans that correspond precisely to the rosettes and whorls seen on histological sections and SD-OCT in *rd7* mice, fundus features are also remarkably similar between ESCS patients and their murine counterpart. The only divergence observed in late disease stages is as follows: in humans, disease typically evolves at and outside the arcades with deepening in the local severity and progressive accumulation of nummular deep pigment deposits vis-à-vis excellent macular sparing in most cases; in mice, late stages are characterized by a progressive fading of the nummular lesions that correspond to the noted rosettes and whorls. This difference is potentially attributable to the lack of a macular region and the different topography and distribution of photoreceptors in murine retinas as compared to human ones.

In this study, we have shown the value of obtaining SD-OCT scans in ESCS patients at the vascular arcades. With the increased availability of wide-field SD-OCT methodologies in the clinic, assessing ESCS patients with such extended scans to include the vascular arcades along both the vertical and horizontal meridians—or, when not yet available, to perform custom scans at the arcades to complement the standard macular ones as we did in this investigation—appears to be highly informative and, thus, advisable. Furthermore, we observed an association between better preserved S-cone mediated retinal function in ESCS patients and specific imaging characteristics by SD-OCT criteria at the vascular arcades. This suggests that there are important functional-microanatomical correlates that could be further exploited to improve our ability to assess change longitudinally in *ESCS* patients, and that it is worth further investigation.

### 4.5. The Value of Perimetric Approaches Focused on S-Cone Function and Their Correlation to Other Perimetric Parameters in ESCS

The importance of utilizing chromatic blue-on-yellow perimetry for the diagnostic confirmation of ESCS has long been established [29,31,32,34]. Herein, we not only demonstrate the parallels in disease modeling that substantiate the use of noninvasive imaging tools such as SD-OCT for early diagnosis, characterization, and follow-up of *NR2E3*-related disease in both patients and preclinical models, but we also confirm in full the value of chromatic perimetry geared towards the assessment of S-cone mediated function in humans. In our hands, comparable to what was reported by Garafalo et al. [38] standard SWAP perimetry already permits such assessment in reliable and useful fashion. Wider testing paradigms replicating this testing methodology across the entire field of vision can also be performed with other commercially available automated perimeters, and appear to be ideal to assess S-cone mediated retinal function also peripherally in ESCS patients. We also specifically highlight how the ring scotomas that characterize the kinetic visual field of subjects with ESCS correspond quite closely to the areas of most severe S-cone sensitivity loss (and, when assessed, even more so to that of L- and M-cones), and how peripheral islands of vision to the III4e target typically predict partial preservation of S-cone mediated vision in those areas. We propose that these criteria can be used to make potential inferences about the extent of residual S-cone function in ESCS patients also when chromatic perimetry data may not be available or be otherwise obtainable.

## 5. Conclusions

In summary, our study not only provides useful correlates of *NR2E3-*associated disease states and progression in patients and the *rd7* mouse to validate the usefulness of the *rd7* mouse model for therapeutic studies, but also identifies imaging and functional features that can be utilized in human studies of ESCS in order to help drive therapeutic agent delivery decisions and identify possible outcome measures in future treatment trials of *NR2E3*-related human recessive disease.

## Figures and Tables

**Table 1:** Demographic and genetic characteristics of affected patients.

	Age	Gender	Ancestry	Visual Acuity		Genotype (NR2E3 Mutations)	
				OD	OS	Allele 1	Allele 2
PT1	9	F	White	20/40 *	20/50 *	IVS-2 A > C (splice site)	c.767C > A, p.Ala256Glu (A256E)
PT2	14	M	Ethipoian	20/20	20/25	c.932G > A, p.Arg311Gln (R311Q)	c.311G > A, p.Arg104Gln (R104Q)
PT3	16	F	Ethiopian	20/25	20/40	c.932G > A, p.Arg311Gln (R311Q)	c.311G > A, p.Arg104Gln (R104Q)
PT4	61	M	White	20/25	20/25	c.932G > A, p.Arg311Gln (R311Q)	c.932G > A, p.Arg311Gln (R311Q)
PT5	69	F	White	20/40	20/30	IVS-2 A > C (splice site)	IVS-2 A > C (splice site)

* High hyperopic refractive defect (amblyopic component). F: female, M: male, OD: right eye, OS: left eye, PT: patient. p.: protein, IVS: inversion. A: adenine, C: cytosine, Arg, R: arginine, Gln, Q: glutamine; Glu, E: glutamic acid.

## Supplementary Materials

The following are available online at https://www.mdpi.com/2077-0383/10/3/475/s1, Figure S1: Examples of normal kinetic visual field and imaging studies. Figure S2: Wide-field SD-OCT findings in PT5; Figure S3: Autofluorescence-OCT macular imaging correlates in PT2; Figure S4: *Nr2e3*-associated Disease Staging Progression; Table S1: Clinical summary of *Nr2e3*-associated disease clinical presentation at time of examination.
