# Supplementary material for: Interspecies Correlations between Human and Mouse NR2E3-Associated Recessive Disease
Source: J Clin Med. 2021 Jan 27;10(3):475. doi: 10.3390/jcm10030475 (PMC7865474; doi:10.3390/jcm10030475)
Supplement: Supplementary file 1 [file jcm-10-00475-s001.pdf]

Journal of
Clinical Medicine

MDPI

Supplementary Material

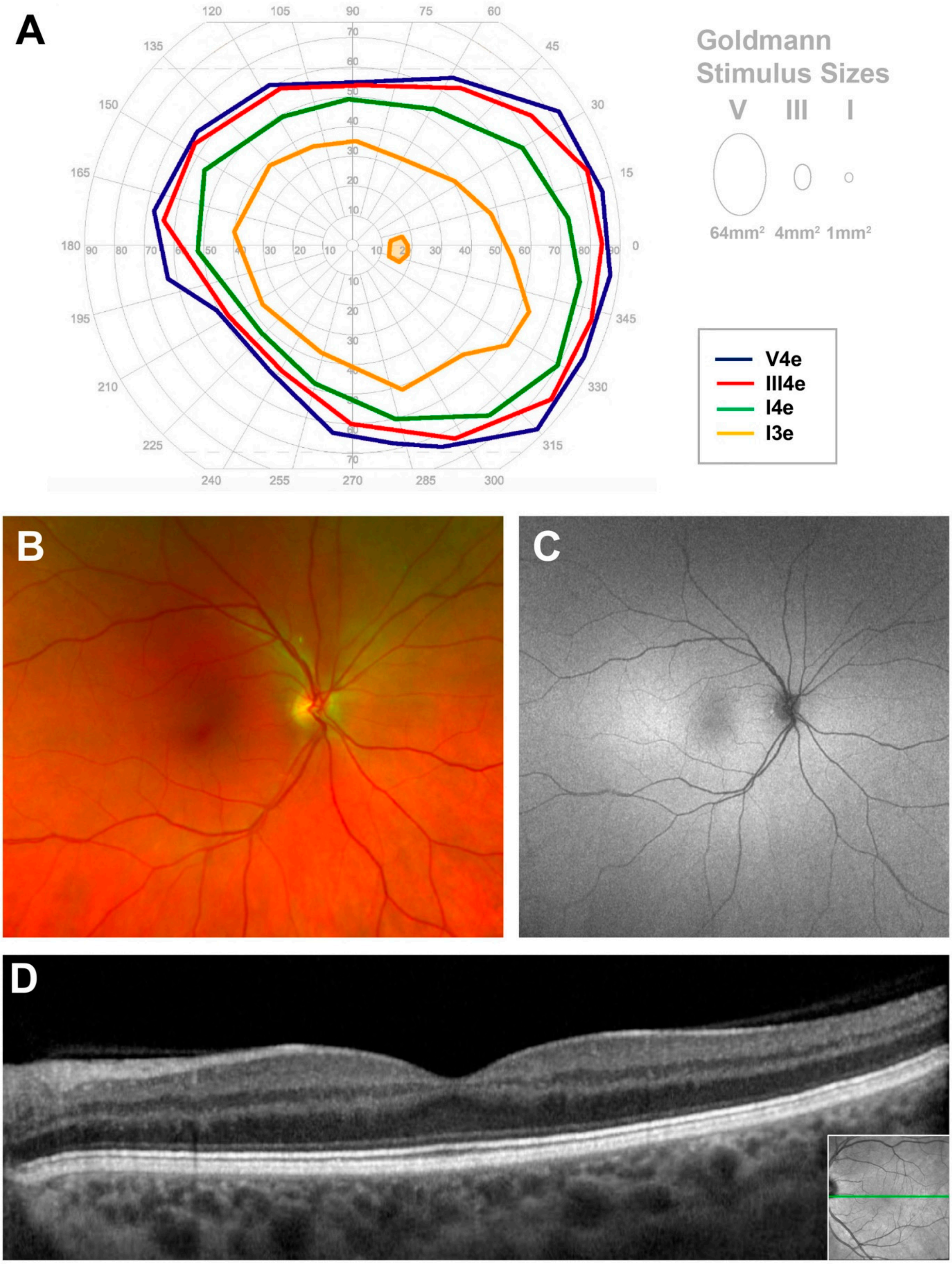

Figure S1. Examples of normal kinetic visual field and imaging studies. **(A).** Example of a normal kinetic visual field (right eye). Each isopter is identified in the color-coded legend. In such a normal example, one would normally be able to delimit also an isopter with a I3e stimulus. This is not customarily possible in retinal dystrophy patients, whose vision quality

does not permit I3e detection in the vast majority of cases. Thus, in the manuscript all fields present only results for the V4e, III4e and I4e targets. Their relative and absolute size is reported in the top right side of this panel. The stimulus brightness is controlled independently by the other parameters. By convention, the "e" parameter is always left unchanged and, if a patient can see stimuli below the I4e, then the brightness (but not the size thereof) is reduced to the "3" level, and so on. **(B).** Example of a normal fundus. **(C).** Example of a normal fundus autofluorescence (FAF). **(D).** Example of a normal spectral domain (SD) OCT scan across the human fovea (central dip in the scan; the scan location is shown in the inset in the bottom lower right corner, green horizontal line). The retinal layers at this location are the same as those seen at any other location, including at the arcades, only thicker at the foveal level than at the arcades.

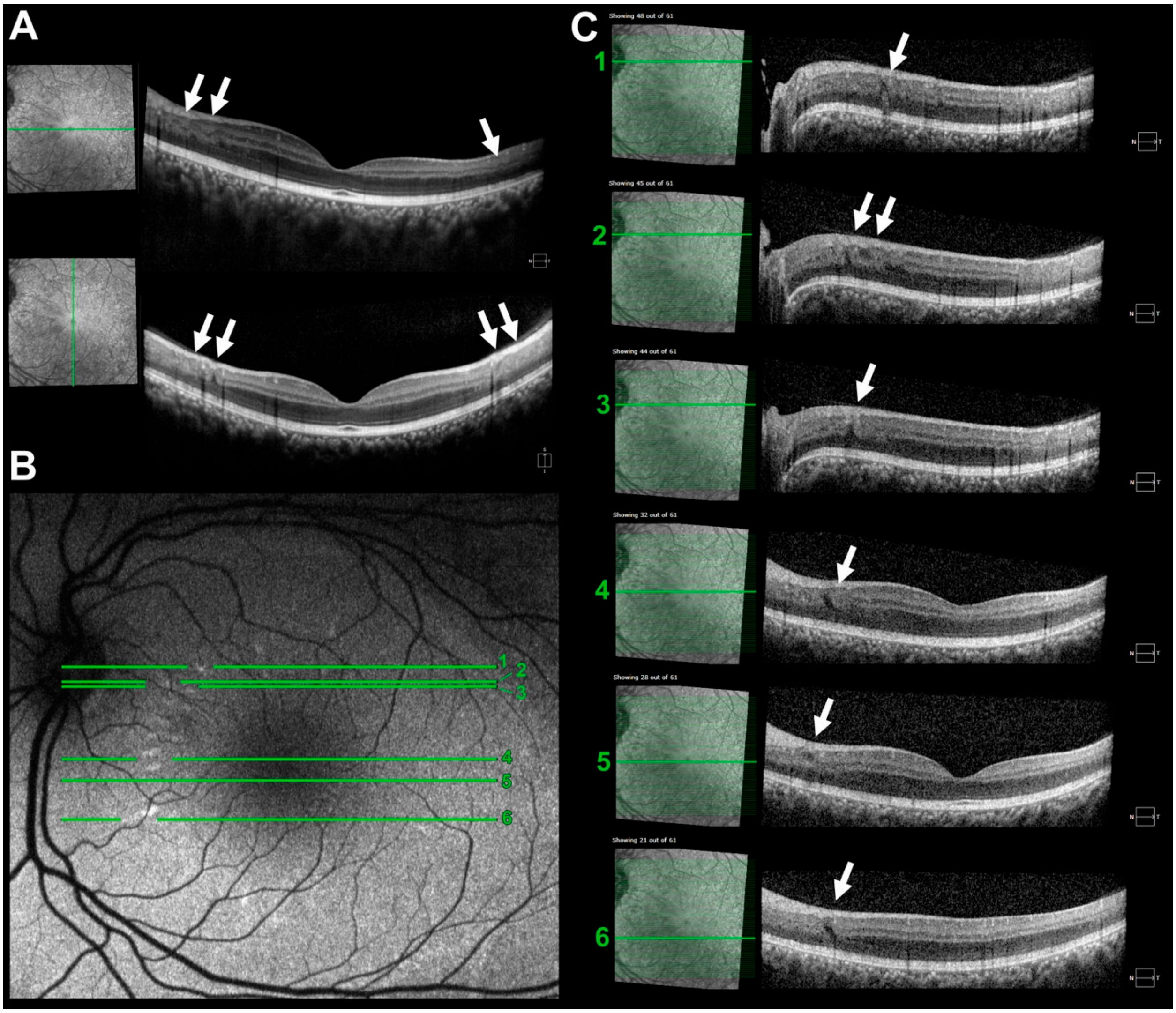

Figure S2. Autofluorescence-OCT macular imaging correlates in PT2. **(A)** Macular SD-OCT findings from the left eye of PT2. The white arrows identify peripheral wavy abnormalities at the level of the ONL, OPL and INL. Note also absence of cystic changes. **(B)** Macular FAF detail from PT2 with overlaid green lines identifying the position of the SD-OCT scans shown in **C**. Except for a single cone-shaped faintly hyperreflective change seen on scan 1 (white arrow), no other discrete, nummular or cone-shaped hyper-reflective change as seen at the arcades in this and all other patients was observed; instead, in PT2, hyper-AF at the level of the RPE appears to coincide mostly, if not exclusively, with intraretinal hypo-reflective spots and cleft-like changes, typically abridging the ONL, OPL and INL (white arrows). No overt underlying hyperreflective change at the level of the RPE level was seen in PT2 corresponding to the faint, linear hyper-AF changes seen on FAF.

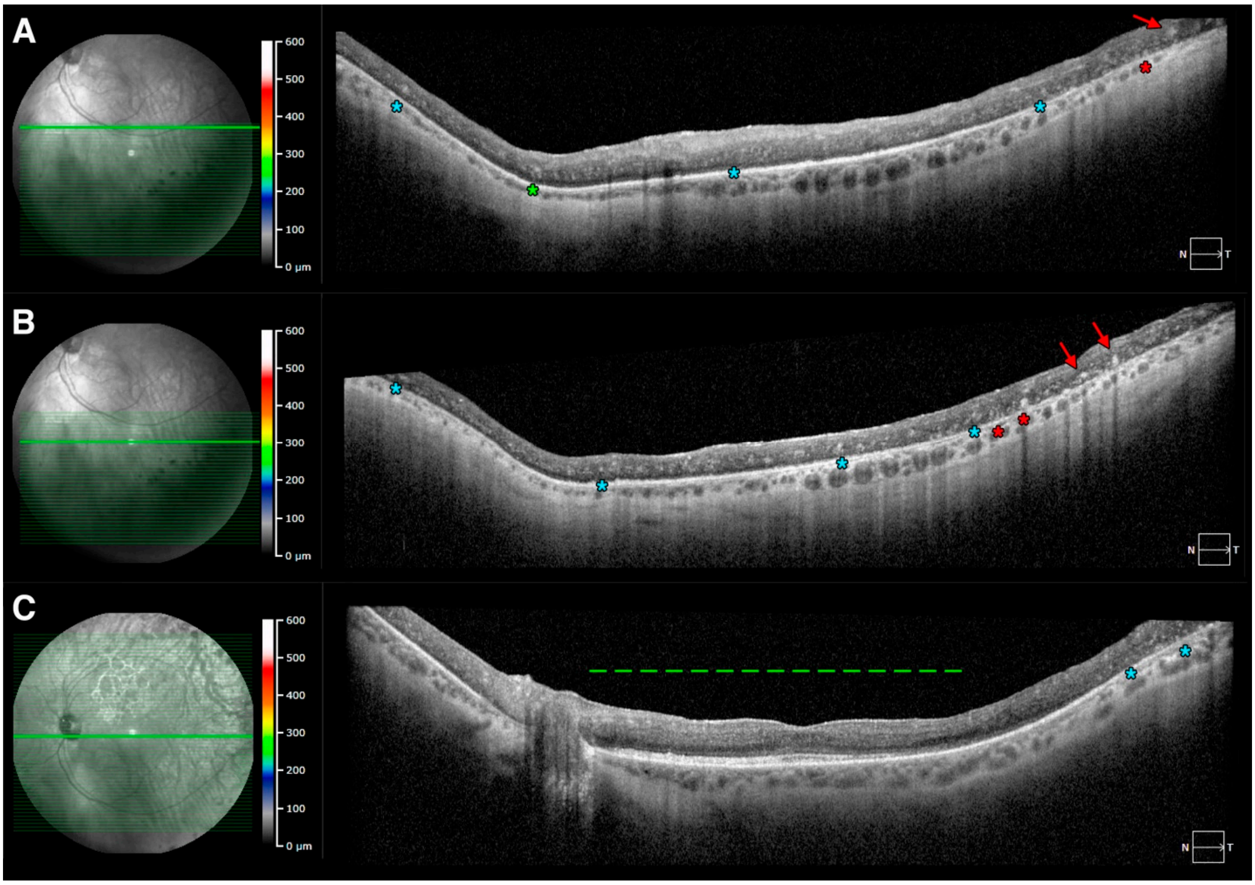

Figure S3. Wide-field SD-OCT findings in PT5. Scans at the inferotemporal arcade in PT5 **(A,B),** which was the least affected and corresponded to areas not exhibiting scotomas on perimetry, presented with fainter, punctate, disseminated irHRFs in the outer retinal third dominated (light blue asterisks) with some occasional better defined nummular lesions (green asterisk, as seen in PT1) and rare far peripheral nummular irHRFs (red arrows). **(C)** Macular trans-foveal scan – The dashed green line identifies the typical width of a macular OCT scan. The optic disc is the columnar structure seen on the scan immediately to the left of the dashed green line. No irHRFs are seen in the central retinal area, and only light blue asterisks type lesions are seen in the far peripheral temporal part of the macular scan (as well as nasal to the optic disc).

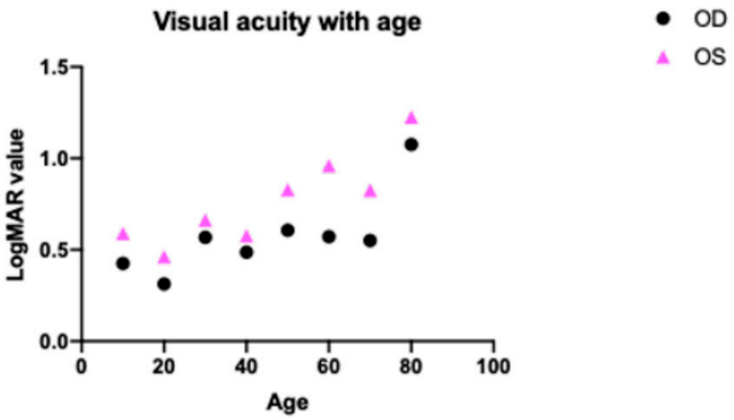

Figure S4. Nr2e3 Disease Staging and Disease Progression. LogMAR plot developed from mean average visual acuity (VA) assessments of over 200 patients' data presented in approximately 30 clinical papers. Data used only where VA was clearly documented. Mean average is grouped by decade to represent right eye (OD) and left eye (OS). Based on available data n= 10 (first decade of life, 0-10 years); n=39 (2nd decade; 11-20 years); n=23 (3rd decade 21-30 years); n=24 (4th decade of life, 31-40 years); n=15 (5th decade, 41-50 years); n=9 (6th decade of life, 51-60 years); n=4 (7th decade of life, 61-70 years); n=8 (8th+ decade of life; 71-88). VA of over 20/200 is represented by LogMAR value of 2. Data generated from patients with confirmed mutations in Nr2e3.

Table S1. Clinical summary of *NR2E3* disease clinical presentation at time of visit subdivided by age strata. This table was generated from review of data from the literature on patients with confirmed mutations in *NR2E3*.

Decade of life	Sample Size (n)	Clinical Presentations/Findings
0-10	26	Night blindness, white spots, intraretinal pigmentation relatively good vision
11-20	55	Night blindness, relatively good visual acuity, white spots, retinoschisis, cysts, some clumped pigment retinal disease, peripheral retinoschisis, ring scotomas
21-30	35	Night blindness, visual field loss, macular cysts, reduced ERG, yellow/white spots along vascular arcade, foveal schisis, nummular pigment deposits in RPE, clumped pigment retinal atrophy
31-40	30	Night blindness, visual field loss, nummular pigment deposits, lesions along arcade, cysts in fovea, retinal/macular atrophy, attenuated retinal vessels, retinal atrophy
41-50	23	Night blindness and mild photophobia, visual field loss, retinal fibrosis, mid peripheral nummular pigment clumping, peripheral pigment clumps, few bone-spicules in periphery, RPE atrophy, bull's eye maculopathy, cystic macular changes, minimal retinal vessel attenuation
51-60	15	Night blindness, variable reduction of ERG, RPE dystrophy along vascular arcade, few spicules, chorioretinal atrophy, outer retinal atrophy with intraretinal pigmentation of mixed nummular and bone spicule type; mild attenuation of retinal vasculature; cataracts
61-70	8	Night blindness, variable reduction of ERG, macular atrophy, clumped retinal pigmentation, intraretinal pigmentation of mixed nummular and bone spicule types, pigment clumps in extreme periphery, Dystrophic RPE, severely attenuated retinal vessels, cataracts
71-88	11	Night blindness, non-recordable ERG, nummular pigmentary lesions, few spicules, RPE atrophy, marked vascular attenuation, cataracts
